# The effect of visual rivalry in peripheral head-mounted displays on mobility

**DOI:** 10.1038/s41598-023-47427-8

**Published:** 2023-11-18

**Authors:** Shui’er Han, Sujin Kim, Jae-Hyun Jung

**Affiliations:** 1 Institute for Infocomm Research, Agency for Science, Technology and Research (A*STAR), Singapore, Singapore; 2 Department of Ophthalmology, Harvard Medical School, Schepens Eye Research Institute of Massachusetts Eye and Ear, Boston, MA USA

**Keywords:** Human behaviour, Motion detection, Object vision, Displays

## Abstract

Recent head-mounted displays and smart glasses use vision multiplexing, an optical approach where two or more views are superimposed on each other. In vision multiplexing, augmented information is presented over an observer’s natural field of view, providing field expansion and critical information during mobility situations like walking and driving. Yet despite its utility, vision multiplexing may produce visual rivalry, a phenomenon where perception alternates between the augmented information and the background scene for seconds at a time. To investigate, we compared the effect of different peripheral vision multiplexing configurations (unilateral opaque, unilateral see-through and bilateral see-through) on the detection of augmented information, incorporating at the same time real-world characteristics (target eccentricity, depth condition, and gaze movement) for a more realistic assessment. Results showed a persistently lower target detection rate in unilateral configurations than the bilateral configuration, suggesting a larger effect of binocular rivalry on target visibility. Nevertheless, this effect does become attenuated when more naturalistic elements are incorporated, and we discuss recommendations for vision multiplexing design and possible avenues for further research.

## Introduction

Vision multiplexing is an optical engineering approach that superimposes augmented information over an observer’s natural field of view^1–3^. It is mainly used to enable visual confusion, where the images of two different objects are presented to the same retinal location^4–6^. This allows us to display multiple sources of information within the given field of view, resulting in field expansion^7^. Vision multiplexing can also produce diplopia (i.e., seeing the same object in two different directions), but unlike visual confusion, diplopia has no application benefit and is typically prevented in vision multiplexing designs^1,3,8^. This paper focuses on visual confusion, although a joint discussion of diplopia and visual confusion in vision multiplexing is presented elsewhere^9^.

Recent head-mounted displays (HMDs) and smart glasses have used two types of vision multiplexing: bi-ocular and monocular vision multiplexing^1^. Earlier HMDs applied bi-ocular vision multiplexing, in which an opaque display panel is mounted onto only one eye (display eye), a design also referred to as a unilateral opaque display. While the opaque panel presents additional information to the display eye, it also occludes part of the background scene. Since the fellow eye is still able to see the occluded part of the background scene by the opaque display, the combination of both eye views in normal binocular vision produces bi-ocular vision multiplexing. Some commercial examples of early bi-ocular vision multiplexing HMDs are the VR1 Monocular (Virtual Realities LLC, Dickinson, TX) and Vuzix M100 smart glasses (Vuzix, Rochester, NY) .

Monocular vision multiplexing can be achieved using new see-through displays^2,10^, which present both the augmented information and the transmitted background scene simultaneously within the field of view. Various forms of unilateral see-through HMDs have been commercialized, including the Eye Tap device^11^ and Google Glass (Google Inc, Mountain View, CA). As these displays are only able to provide monocular augmented information, bilateral see-through HMDs (i.e., see-through displays mounted on both eyes) were recently developed to present stereoscopic augmented information on top of the background scene. These forms of HMDs include the Microsoft Hololens (Microsoft, Redmond, Washington), Magic Leap (Magic Leap, Inc., Plantation, Florida), and Epson Moverio (Epson America, Inc., Long Beach, CA), which respectively provide capabilities for mixed and augmented realities with binocular disparities.

Apart from commercial HMDs, vision multiplexing has also been applied clinically to provide field expansion in patients with visual field loss. For example, a patient who has lost part of the visual field on both eyes (e.g., homonymous hemianopia or tunnel vision) may be fitted with unilateral prisms, which superimposes the prism-shifted parts of the blind fields and the normal view onto the residual visual fields with bi-ocular vision multiplexing^7,12,13^. When the field loss results in no binocular vision in the residual field, the patient can be fitted with multiplexing prisms (i.e., see-through prism) that enable monocular visual confusion^2,3,8^. In both forms of vision multiplexing, the prisms superimpose the scene from the blind field of the patient onto the seeing field as visual confusion, which results in true field expansion^7^.

The capability to provide multiple sources of information within the same field of view allows vision multiplexing to be useful in mobility situations such as walking and driving. For commercial multiplexing HMDs including head-up displays in vehicles, critical hazards and safety information can be presented near the user’s line of sight, allowing the user to maintain their gaze and attention on the road or sidewalk^14,15^. Commercial multiplexing HMDs can also aid aircraft or helicopter landings in adverse weather conditions with low visibility, such as during a fog or heavy rain^16,17^. In clinical populations, patients with field loss often report collisions with other oncoming hazards from their blind field in mobility^18^. By applying vision multiplexing to the field expansion device (i.e., multiplexing prisms), the visual information from the blind field can be rendered to the residual visual field. These true field expansion devices have been used to alert the hazards from the blind field, thus improving their driving^19–21^ and walking performances^8,13^.

Despite the utility of vision multiplexing, it may lead to unintended perceptual experiences such as visual rivalry^22,23^. In bi-ocular vision multiplexing, the content within the display’s field of view on the display eye conflicts with the corresponding part of the scene in the fellow eye. This may provoke *binocular rivalry*, a phenomenon where visual perception alternates between the different images on each eye (i.e., augmented information in the display eye and the background image in the fellow eye) every few seconds^24^. In contrast, monocular vision multiplexing displays such as unilateral and bilateral see-through HMDs may produce *monocular rivalry* within the display eye(s). Like binocular rivalry, visual perception alternates between the superimposed augmented image and the background scene, but with a less salient appearance and at a slower rate^25^. A key difference between unilateral and bilateral see-through HMDs is that the former configuration is also susceptible to binocular rivalry, triggered by the conflict between the superimposed image composite (i.e., see-through augmented information and background) in the display eye and the background scene in the fellow eye.

During visual rivalry, the augmented information will be suppressed from awareness when the background image is dominant, even though the information is clearly displayed to the user wearing the HMD. A viable solution is to conduct peripheral fitting of multiplexing devices, as studies have reported a slower rate of rivalry alternations^26^ and more tolerance to diplopia^27,28^ in the periphery. There are already some existing peripheral multiplexing HMDs like the Google Glass (Google Inc, Mountain View, CA) which mounts a see-through display at 7$^\circ$ in the upper periphery^29^, and field expansion peripheral prisms that are fitted about 20$^\circ$ in the upper and lower peripheries^2,7,13,30^. However, the effect of visual rivalry may still persist even in these peripheral display devices, as a recent study by Shen et al.^31^ found only about 50% target detection with unilateral opaque displays at 20$^\circ$ vertical eccentricity, specifically with a moving background.

If visual rivalry persists in the periphery with moving backgrounds, then uses of vision multiplexing HMDs for mobility may be implicated. Since different vision multiplexing configurations could provoke binocular and/or monocular rivalry, the visibility of the peripheral multiplexing displays could depend on the type of multiplexing (i.e., unilateral opaque and see-through and bilateral see-through displays). Shen et al.^31^ indicated a substantial effect of binocular rivalry on target visibility in the peripheral unilateral opaque display. While a similar effect of binocular rivalry is expected in the unilateral see-through display, an additional influence of monocular rivalry is yet to be explored. More recently developed peripheral bilateral see-through displays may completely avoid binocular rivalry, but the display visibility may still be compromised by monocular rivalry. To our best knowledge, target detection in the bilateral peripheral see-through configuration has not been tested and compared with other configurations, much less examined during mobility. It is therefore unclear if target detection would be similarly affected by monocular rivalry in this configuration, though there is some evidence showing better detection rates with a central bilateral see-through display^32–34^. To investigate, we compared the effect of different peripheral vision multiplexing configurations (i.e., unilateral opaque, unilateral see-through and bilateral see-through) on the detection of augmented information. In addition, we incorporated real-world characteristics (eccentricity of the peripheral displays, depth condition, and gaze movement) into three different experiments.

## Methods

### Subjects

A total of 19 subjects (age range: 23 years to 51 years) were recruited, three of which are the authors. Of these 19, 12 participated in Experiment 1, and 13 each in Experiment 2 and 3. The overlap of participants across experiments was solely due to their availability. All subjects had normal or corrected-to-normal visual acuity (better than 20/25) and normal stereo vision (better than 30 arcsec in the Randot Stereotest). Three authors participated in Experiments 1 and 2, and two (SK and JJ) took part in Experiment 3. Since excluding the authors’ data did not significantly change the trend of the results, we included data from authors. Informed consent was obtained from all subjects. The experiments conducted in this study adhered to the Declaration of Helsinki and were approved by the Massachusetts Eye and Ear Human Studies Committee.

### Common stimuli

**Figure 1:**
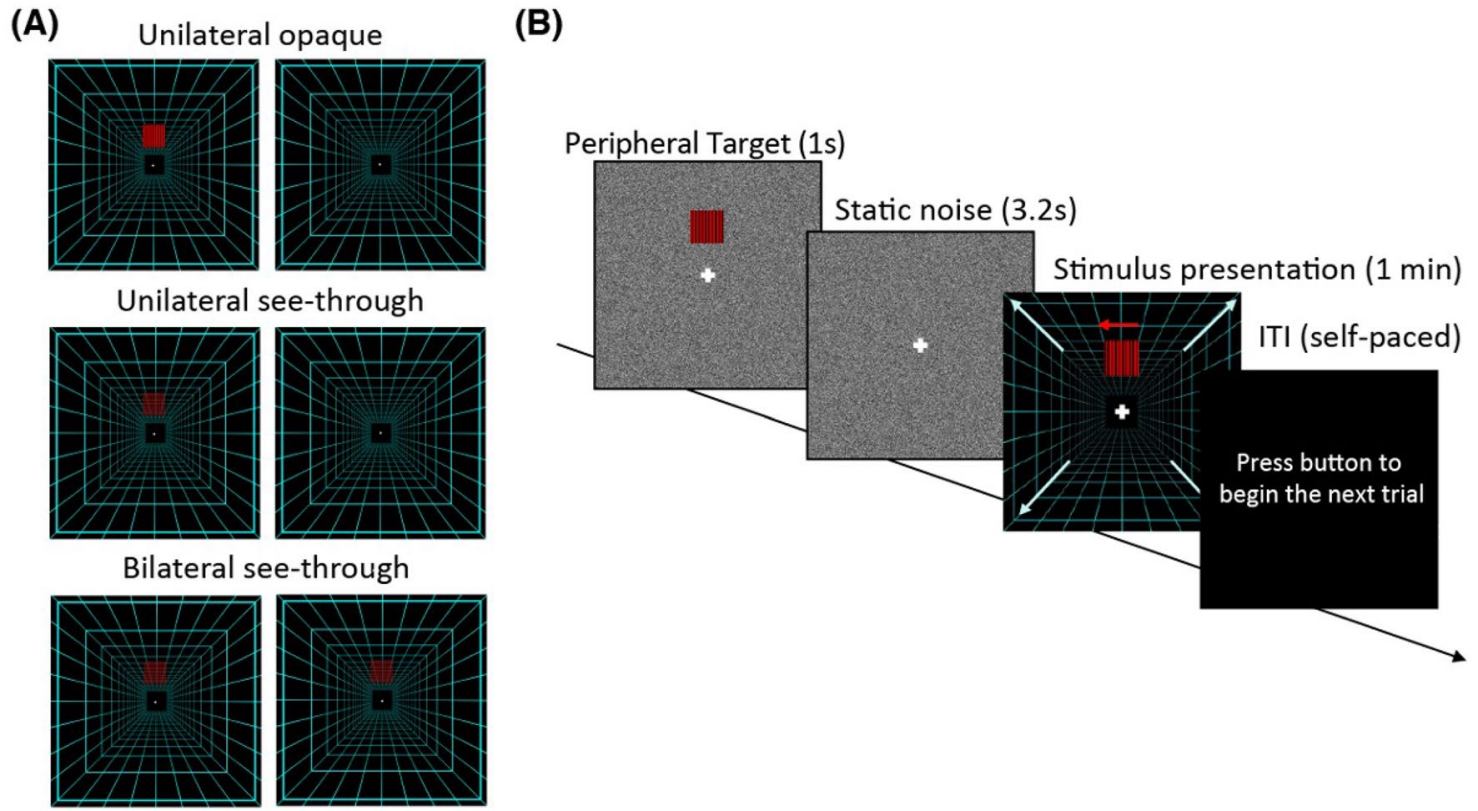
Experimental setup. (**A**) The types of multiplexing configurations tested in this study (left and right eye views). The peripheral target is presented to only one eye for the unilateral opaque and see-through conditions, varying only in the transparency of the peripheral target. Peripheral targets were presented to both eyes in the bilateral see-through configuration, and were set to 50% transparency for see-through configurations (see Video S1). (**B**) Experimental procedure. At the beginning of each trial, a peripheral target was briefly presented (1 s) to indicate its location on a random noise background with a central fixation point. After the peripheral target disappeared, the background remained on a screen for another 3.2 s. Then a forward-moving background (denoted by the diagonal white arrows) and a horizontally drifting (represented by the leftward red arrow) peripheral target (i.e., simulated peripheral display) were presented for 1 min. Subjects were asked to report whenever a third or more of the peripheral target is suppressed by releasing a button on a controller.

Figure 1 shows the common stimuli we used for the experiments. To simulate the optic flow of forward walking, we created a virtual corridor in the Unity game engine (Unity Technologies, San Francisco, USA). The corridor was composed of perspective lines (1 m horizontal spacing), 8 m $\times$ 8 m squares (2 m spacing in depth), and was 50 m in length (Fig. 1A). Forward walking was simulated by moving the corridor at a constant adult walking speed of 1.4 m/s^35^.

The different peripheral multiplexing configurations were each simulated with a 10$^\circ$ by 10$^\circ$ peripheral target, which was larger than the target sizes used in previous studies (e.g., 0.7$^\circ$ static targets in Winterboom et al.^32^). This choice ensured that our observations were more representative of any real-world visual rivalry effects, as smaller targets are generally more strongly suppressed during binocular rivalry^26^. The peripheral target was also a horizontally drifting grating with a spatial frequency of 1 cycle per degree and a temporal frequency of 3 Hz. Image features such as spatial frequency and temporal frequency are known to influence the visibility of the object during visual rivalries^36–38^. In this study, we chose the spatiotemporal parameters that corresponded to high contrast sensitivity in the periphery^39,40^ and mimicked practical augmented information in currently available multiplexing displays. For example, the clock digit height of the Google Glass display is about a third of the display height (i.e., about 2.4$^\circ$ out of 7.3$^\circ$) which gives an estimated font weight of about 1 cycle per degree for the character E. The different motion direction of the grating and its speed (i.e., 3$^\circ$/s, about 1.58 m/s) simulate the optic flow differences with field expansion prisms and faster optic flow signals from the side of the moving corridor^41^.

During the experiment, the peripheral target was presented above the fixation point, visible to one eye in the unilateral opaque and see-through configurations, or both eyes in the bilateral see-through configuration. Targets were set to an alpha level of 1.0 for the unilateral opaque configuration, and an alpha level of 0.5 for both see-through configurations.

### Common procedure

Figure 1B describes the experimental procedure for one trial. Each trial began with the presentation of the central fixation cross on a random noise image (82$^\circ$ by 82$^\circ$). A static frame of the peripheral target was presented for 1 s and cued the peripheral target location. The random noise remained on the screen for 3.2 s to minimize any potential color and motion after-effects. Following that, the peripheral target (i.e., simulated peripheral display) was presented on the simulated walking corridor for 1 min. Subjects were instructed to hold down the controller button and release it whenever a third or more of the target disappeared (i.e., partial suppression of the target; see^42^). This criterion was chosen because it represented a significant missing portion of augmented information (e.g., missing at least two clock digits on the Google Glass).

To allow subjects to become accustomed with the task demands, a practice session of 24 trials was conducted prior to the main experiment. Each practice trial presented a 2D video of the moving background and the peripheral target. However, rivalry suppression of the target was mimicked in the practice trial (Video S1), such that parts of the target gradually faded into a mixture with the moving background. Here, subjects were asked to press and hold the button to initiate each trial and release it as soon as more than a third of the target disappeared. Each trial ended with the button release and no feedback was provided about the actual target visibility.

### Experiment 1: effects of multiplexing configuration and eccentricity on target visibility

As the effect of eccentricity on target detection in multiplexing HMDs has not been systematically explored, we varied target eccentricity over a range that covers current peripheral display locations (5$^\circ$, 10$^\circ$, and 15$^\circ$), resulting in a 3 $\times$ 3 factorial design with multiplexing configuration and target eccentricity as independent variables. Experiment 1 examined the different multiplexing configurations under more typical laboratory conditions employed in the previous studies^31–33,43^. Thus, the moving corridor was recorded as a 2D video (82$^\circ$ by 82$^\circ$ field of view, 1 min duration) with Unity’s inbuilt video recorder, presented on a virtual screen of matching size at 30 m in depth and viewed via the full fields of Meta Quest 2 (Meta Platforms, Inc.) HMD. Fixation was maintained using a central cross (0.7$^\circ$ $\times$ 0.7$^\circ$) located at the same distance.

Target eccentricity was tested separately in 3 different blocks (i.e., within a block, target location was fixed at either 5$^\circ$, 10$^\circ$ or 15$^\circ$ from the fixation point), and each block presented 2 trials of each multiplexing configuration. The target eccentricity was maintained the same within each block to minimize attentional effect on its visibility owing to continuous changes in target location. The presentation of multiplexing configurations was fully randomized within each block.

### Experiment 2: effects of multiplexing configuration and depth condition on target visibility

When augmented reality HMDs are used during real-world mobility, the background varies continuously in depth across eccentricities. This produces various depth differences between the display and background, depending on where the user is fixating within the display. To investigate its effect on the same three multiplexing configurations, Experiment 2 compared the target visibility of each configuration in a 3D moving background (i.e., continuously varying depth condition) with that of a 2D moving background (i.e., single depth condition). The 2D recorded corridor from Experiment 1 was presented in the 2D condition, whereas the moving 3D corridor was used for the 3D condition. Thus the 3D condition contained continuously varying stereoscopic depth cues of the 3D corridor (i.e., farther eccentricity had closer depth), which was provided on the full fields of Meta Quest 2. Additionally, the peripheral target and fixation point were located at 1 m and 30 m, respectively. This simulates the depth differences that would have occurred between a real-world multiplexing HMD and the user’s gaze point during mobility, where the user will generally look further ahead in the environment and view the display through peripheral vision or sporadic glimpses. Note that the stereoscopic depth cues of the target could only be perceived in the bilateral see-through condition (binocular viewing), but not in the monocular conditions (monocular viewing). As we found a similar trend of multiplexing configuration across eccentricities in Experiment 1 (see “Results” section), the target eccentricity was fixed at 10$^\circ$. The presentation of the depth conditions and multiplexing configurations were fully randomized across 2 blocks of 6 trials, allowing 2 trials for each combination of multiplexing configuration and depth condition.

### Experiment 3: the effect of multiplexing configuration and eye movement on target visibility

In addition to depth differences between the HMD and background in real-world mobility situations, users tend to make eye movements as they scan the external environment. To test the effect of eye movements on target visibility in different multiplexing configurations, we used the same 3D depth condition and peripheral targets (horizontal drifting grating at 10$^\circ$ eccentricity) from Experiment 2. The same fixation cross (30 m from the subject) was used to guide three different eye movement conditions (central fixation, saccadic eye movement, and smooth pursuit). In the central fixation condition, the fixation cross remained in the center of the screen throughout the whole experiment. For saccadic eye movement and smooth pursuit conditions, a range of fixation cross locations was first defined, creating a bounding box that extended ± 2.5$^\circ$ horizontally and ± 1.5$^\circ$ vertically from the center of the corridor. In the saccadic eye movement condition, the fixation cross randomly jumped to any location within the bounding box at every 5 s. This differed from the smooth-pursuit condition, where the fixation cross moved smoothly at 7.1$^\circ$/s laterally and 2.1$^\circ$/s vertically within the bounding box (see Video S1). Eye movements were verified using the eye tracking capability of the HTC VIVE Pro Eye (HTC Corporation, New Taipei City, Taiwan) HMD. Eye tracking was implemented through a software development kit (SDK) provided by HTC Corporation (SRanipal), and eye movements were recorded at a sampling frequency of 90 Hz. For each time point, we recorded the validity and openness of each eye and position, and gaze origin and direction from the data structure provided in SRanipal SDK (ViveSR.anipal.Eye.EyeData_v2). In addition, an amount of gaze error was calculated real-time as the Euclidean distance between the locations of fixation point and gaze. Similar to Experiments 1–2, subjects were required to report the target visibility with a button press. In addition to the target visibility, subjects were also required to track the location of the fixation cross to simulate different types of eye movements. The type of eye movement and the different multiplexing configurations were fully randomized across 3 blocks of 6 trials (i.e., 2 trials per multiplexing configuration). To ensure the precision of eye tracking, eye movements were calibrated at the beginning of each block.

### Data analysis

Target visibility was defined as the percentage of total viewing time where subjects reported seeing approximately two-thirds or more of the area of the peripheral target. For each experiment, the effects of multiplexing configuration and the factor of interest (i.e., eccentricity in Experiment 1, background type in Experiment 2, and eye movement in Experiment 3) on target visibility were analyzed by two-way repeated measures ANOVAs, corrected with the Greenhouse−Geisser method whenever data normality was violated. Post-hoc paired samples t-tests were conducted where applicable, and the results were corrected with the Holm–Bonferroni method. In Experiment 3, the collected eye movement dataset was screened for invalid (e.g., eye blinks) and outlier (> 3 standard deviations from the mean) data points. These data points were excluded from further analyses and the mean gaze error per trial was calculated with the remaining data. Eye movement data from two subjects were excluded due to the technical issues during data acquisition.

## Results

### Experiment 1: effects of multiplexing configuration and eccentricity on target visibility

**Figure 2:**
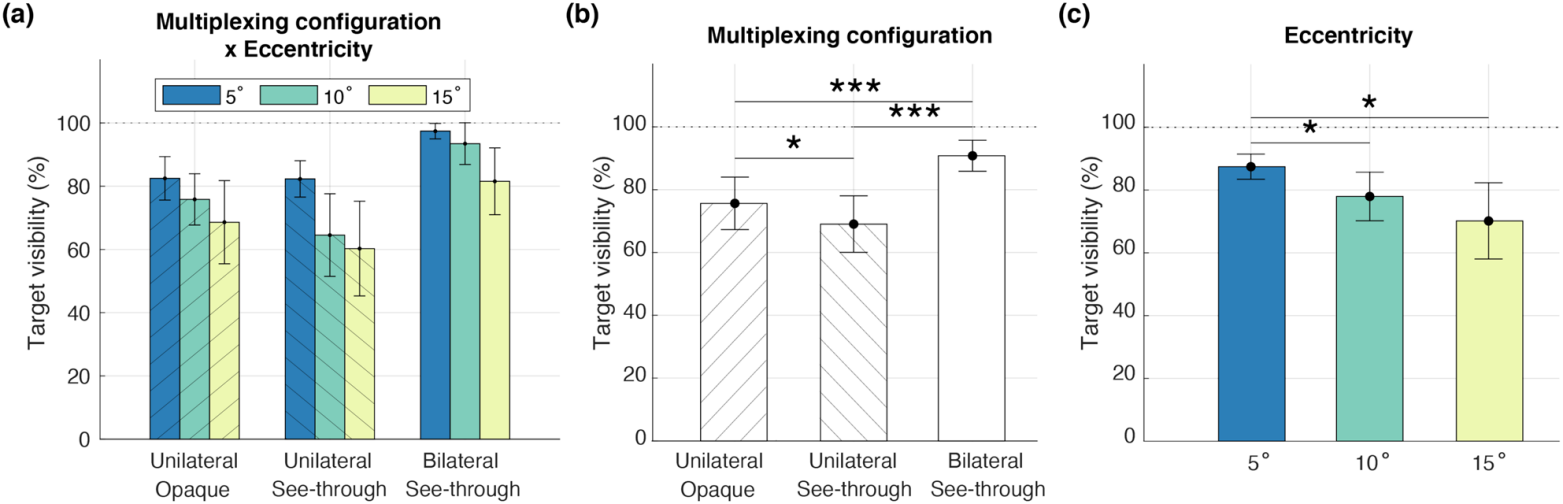
Effects of multiplexing configuration and eccentricity on peripheral target visibility. (**a**) Interaction between the multiplexing configuration and eccentricity. (**b**) Main effect of multiplexing configuration: Unilateral opaque, Unilateral see-through, and Bilateral see-through. The bilateral see-through configuration yielded greater target visibility than both unilateral opaque and unilateral see-through configurations. (**c**) Main effect of eccentricity. Visibility of the target is reduced when it is located farther periphery. All error bars indicate 95% confidence intervals. Asterisks represent statistical significance ($$*p< 0.05; {**}p< 0.01;{***}p<0.001$$), and all p-values were corrected with the Holm–Bonferroni method for multiple comparisons.

We found a significant interaction between target eccentricity and multiplexing configuration (Fig. 2a; $F(4,44) = 3.66, p = 0.012, \eta ^2 = 0.25$), characterized by a larger decrease in target visibility in unilateral multiplexing configurations. When target visibility was compared between pairs of varying eccentricity conditions, we observed an significantly higher visibility at the eccentricity of 5$^\circ$ than 10$^\circ$ ($t(11) = 3.22, p = 0.019, d = 0.93$) and 15$^\circ$ ($t(11) = 3.36, p = 0.019, d = 0.97$). This is in accordance with the previous literature that showed a decrease in visual task performance with increasing eccentricity^44–46^.

Target visibility was significantly modulated by both multiplexing configuration (Fig. 2b; $F(2,22) = 28.02, p < 0.001, \eta ^2 = 0.72$) and eccentricity (Fig. 2c; $F(2,22) = 7.96, p = 0.003, \eta ^2 = 0.42$). For multiplexing configuration, post hoc pairwise comparisons showed that the bilateral see-through target had a significantly higher visibility than the unilateral opaque ($t(11) = 5.11, p < 0.001, d = 1.5$) and unilateral see-through ($t(11) = 6.01, p < 0.001, d = 1.7$) targets. This may suggest a significant effect of binocular rivalry on the visibility of these multiplexing configurations. Furthermore, the unilateral target was more visible when it was opaque compared to when it was see-through ($t(11) = 3.03, p = 0.011, d = 0.87$). While it may suggest suppression of the target by monocular rivalry between the augmented foreground and the see-through moving background, in addition to the binocular rivalry, the reduced contrast of the target may have also contributed to the lower target visibility. But since the visibility of the bilateral see-through target was still higher than the unilateral opaque target, despite being of lower contrast, the contribution of binocular rivalry to target visibility may be more significant than target contrast.

### Experiment 2: effects of multiplexing configuration and depth condition on target visibility

**Figure 3:**
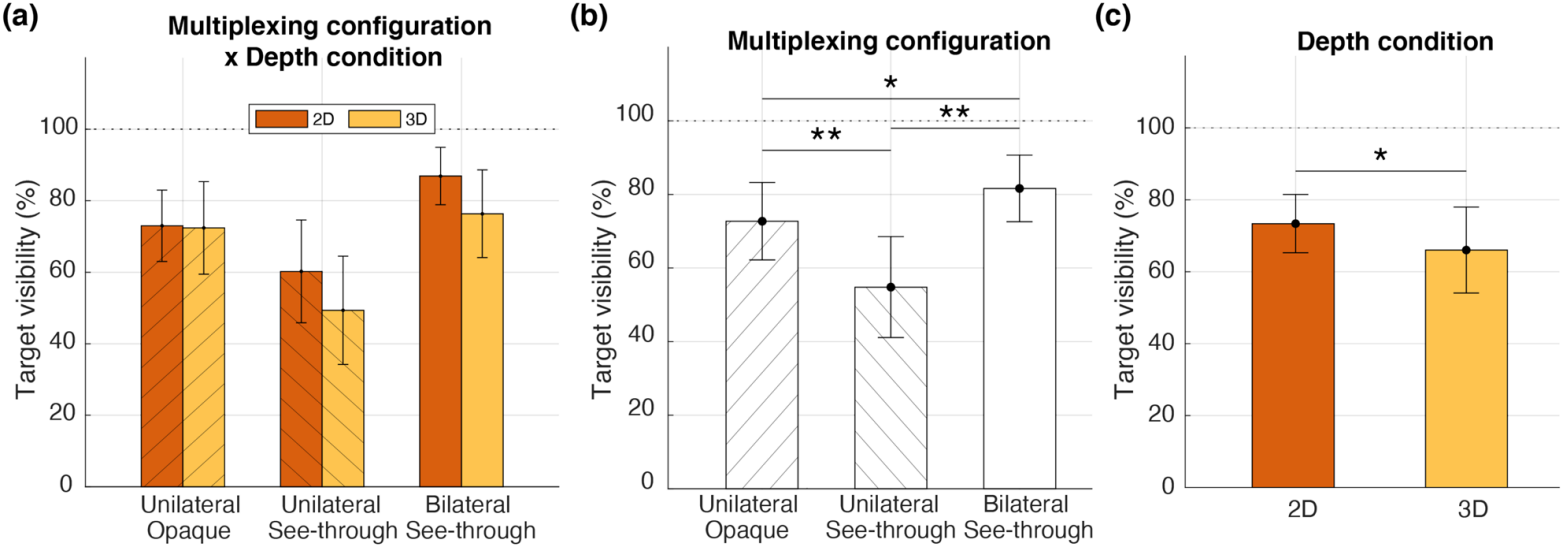
Effects of multiplexing configuration and depth condition on peripheral target visibility. (**a**) Interaction between the multiplexing configuration and depth condition. In see-through configurations, the target visibility in the 3D depth condition was lower than that in the 2D depth condition. (**b**) Main effect of the multiplexing configuration. (**c**) Main effect of the depth condition.

Background depth condition did not interact with multiplexing configuration (Fig. 3a; $F(2,24) = 0.65, p = 0.21, \eta ^2 = 0.121$), but we observed a significant main effect of multiplexing configuration on target visibility (Fig. 3b; $F(2,24) = 18.18, p < 0.001, \eta ^2 = 0.6$). As in Experiment 1, the bilateral see-through configuration had a higher target visibility than the unilateral opaque ($t(12) = 2.36, p = 0.036, d = 0.65$) and unilateral see-through configurations ($t(12) = 4.62, p = 0.001, d = 1.28$), suggesting again that binocular rivalry, not target contrast, may have a larger limiting effect on multiplexing display functionality. The target visibility was also lower in the unilateral see-through configuration compared to the unilateral opaque configuration ($t(12) = 4.85, p = 0.001, d = 1.35$), presumably due to the lower target contrast and the monocular rivalry between the see-through target and background.

Target visibility also depended on the depth condition (Fig. 3c; $F(1,12) = 6.42, p = 0.026, \eta ^2 = 0.35$), becoming less visible when the bilateral see-through configuration is presented with the 3D depth condition ($t(12) = 2.27, p = 0.043, d = 0.63$). The unilateral see-through configuration was also less visible in the 3D condition than the 2D condition, but the difference was not statistically significant ($t(12) = 2.16, p = 0.052, d = 0.6$).

### Experiment 3: the effect of multiplexing configuration and eye movement on target visibility

**Figure 4:**
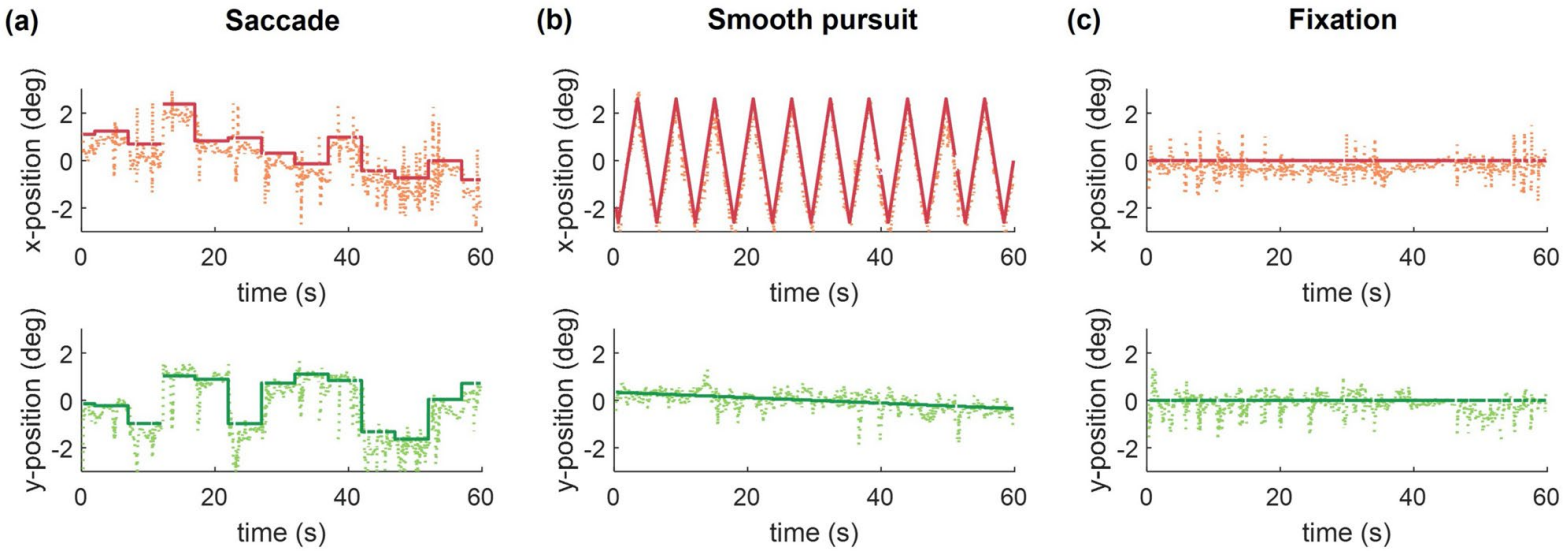
Example of fixation cross positions (solid lines) and subject’s gaze positions (dotted lines) in one trial for each eye movement condition. Data were derived from a representative subject. Note that the discontinuation in the lines is due to the elimination of invalid data (e.g., blinks) (**a**) Saccade condition. Horizontal and vertical positions of the fixation cross were randomly changed within the predefined boundary every 5 s. (**b**) Smooth-pursuit condition. The fixation cross moves back and forth laterally within the predefined boundary, changing its vertical position upwards or downwards when the fixation cross reaches the boundary. (**c**) Central fixation condition. The fixation cross stayed at the center of the corridor throughout the trial.

We first verified that subjects performed eye movements as instructed. Examples of fixation position and subject’s gaze across time in one trial are shown in Fig. 4a–c for each eye movement condition. Mean gaze error for all subjects was $1.54^\circ$ (range $= 0.82^\circ -2.04^\circ$), and the amount of error was slightly smaller for fixation condition ($1.29^\circ \pm 1.23^\circ$, 95% CI) compared to both saccade ($t(10) = 2.37, p = 0.039$; $1.67^\circ \pm 0.35^\circ$) and smooth pursuit ($t(10) = 2.61, p = 0.026$; $1.68^\circ \pm 0.34^\circ$) conditions.

**Figure 5:**
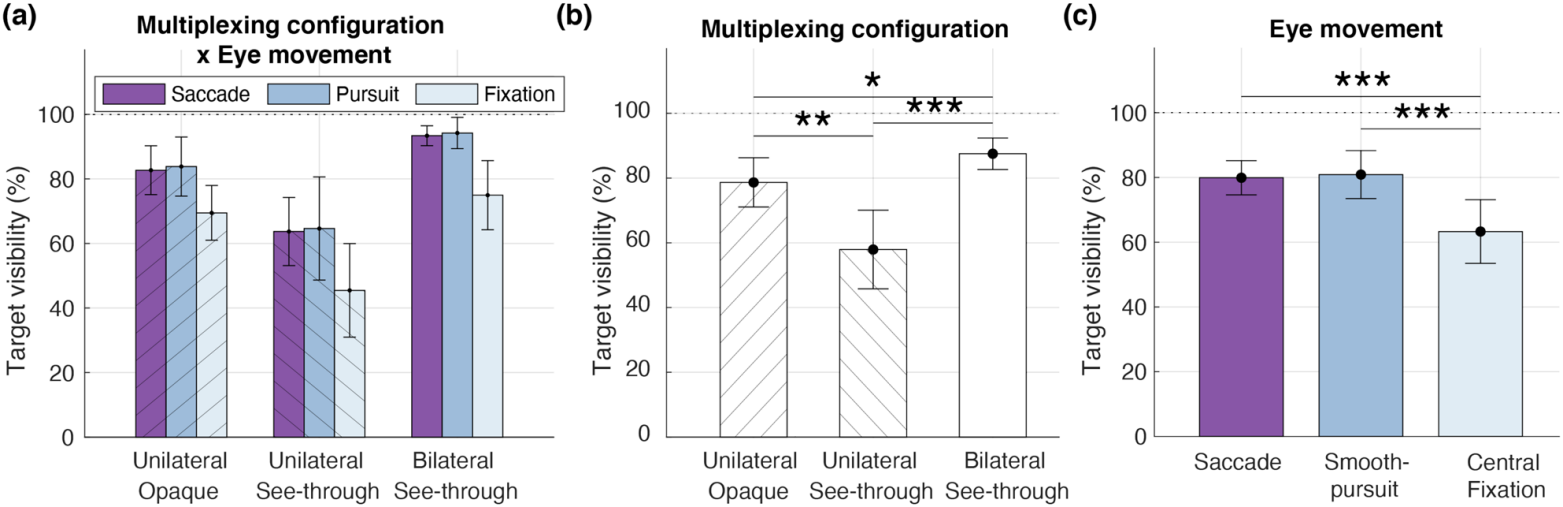
Effects of multiplexing configuration and eye movement on target visibility. (**a**) Multiplexing configuration and eye movement condition did not have a significant interaction effect on target visibility. (**b**) Main effect of multiplexing configuration. (**c**) Main effect of eye movement. The target was more visible when saccadic or smooth-pursuit eye movements were executed, compared to when fixating at the central location.

Having verified the guided eye movements, we proceeded to conduct statistical analysis on the behavioral data. The interaction between multiplexing configuration and eye movement was not statistically significant ($F(2.01,24.15) = 0.4, p = 0.675, \eta ^2 = 0.03$). There were, however, significant main effects of multiplexing configuration (Fig. 5b; $F(2,24) = 24.45, p < 0.001, \eta ^2 = 0.67$) and eye movement (Fig. 5c; $F(2,24) = 23.17, p < 0.001, \eta ^2 = 0.66$) on target visibility. Similar to the findings from Experiment 1 and 2, the bilateral see-through target was most visible (Fig. 5b), and the unilateral opaque target was more visible than the unilateral see-through target ($t(12) = 3.98, p = 0.004, d = 1.1$). When target visibility was compared across three eye movement conditions, results showed that the target was more visible when executing eye movements (saccade versus fixation: $t(12) = 5.86, p < 0.001, d = 1.63$; pursuit versus fixation: $t(12) = 5.04, p < 0.001, d = 1.4$). There was no significant difference between the saccadic and smooth pursuit eye movements $t(12) = 0.43, p = 0.676, d = 0.12$).

## Discussion

We investigated how different vision multiplexing configurations affect the detection of augmented information during mobility. Three types of multiplexing display configurations (i.e., unilateral opaque, unilateral see-through, and bilateral see-through displays) were tested in the periphery, each with the potential of provoking monocular rivalry and/or binocular rivalry. The configurations were simulated using peripheral moving targets, presented in a 3D walking simulation created in VR. This is a distinction from previous studies, which had not evaluated target detection in peripheral bilateral see-through displays (e.g., Hololens and Magic Leap) and whose stimulus conditions did not realistically capture mobility (e.g., 2D backgrounds in Refs.^31,32^). Unlike many central visual rivalry studies that tracked the visibility of both competing images^22^, we measured the visibility of the augmented target. This was because the background scene is always visible in real-world applications, either outside the display field of view or within a see-through display. Tracking the visibility of the augmented target is apt, as the target is only detectable when it is not substantially suppressed (i.e., a third or more in this paper).

Previous work have suggested that binocular rivalry has a larger effect on target detection than monocular rivalry^32^. Consistent with this proposal, we found that both unilateral multiplexing configurations were less visible than the bilateral see-through configuration, which remained visible for more than 80% of the viewing time regardless of target eccentricity (Fig. 2, Experiment 1), depth condition (Fig. 3, Experiment 2) and eye movement type (Fig. 5, Experiment 3). These findings demonstrate a persistent effect of binocular rivalry in the periphery during mobility, including for the first time, a significantly smaller effect of monocular rivalry in the peripheral bilateral see-through configuration. Yet when both forms of visual rivalries are involved, as is the case for the unilateral see-through target, overall visibility drops an additional  20% (Figs. 2, 3, 5). This further decrease is partly attributable to the perceptual switches between the background motion and semi-transparent target during monocular rivalry, but it could also stem from the lower monocular contrast of the see-through target, which enhances binocular rivalry suppression^47^. Although the bilateral see-through target too has a lower monocular contrast, the suppression of the target may not be synchronized between the eyes. If at any point, one of the double images is more suppressed by monocular rivalry, subjects will still be able to detect the target from the less suppressed image, thereby contributing to the higher target visibility. Target visibility may also be affected by the binocular summation of monocular see-through images^48^. In contrast, the unilateral targets do not contain stereoscopic depth cues and do not benefit from this support. Additionally, because the bilateral see-through configuration (monocular rivalry with lower contrast) resulted in higher target visibility than the unilateral opaque configuration (binocular rivalry with higher contrast), it further demonstrates how binocular rivalry may contribute more significantly to the visibility than contrast reduction.

Nevertheless, despite the larger effect of binocular rivalry than monocular rivalry in our results, its effect on the visibility of unilateral targets was substantially attenuated than previous reports. Across all experiments, the detection rate of the unilateral opaque target during central fixation was about 70%. This is a lot larger than that observed in Shen et al.^31^, who observed a detection rate of about 38% when normally-sighted participants with simulated left hemianopia were fitted with a peripheral unilateral opaque display and tested with central fixation. Part of this difference could be attributed to the different eccentricity used (i.e., furthest eccentricity is 15$^\circ$ in our study compared to the 20$^\circ$ used in their study), but it could also be the result of using larger, more realistic target sizes in the current study (i.e., 10$^\circ$ by 10$^\circ$ target versus 1$^\circ$ by 1$^\circ$ target in Ref.^31^). Unlike smaller targets, larger target sizes tend to produce partial suppression^42^. By requiring subjects to use a more realistic criteria such as partial visibility (i.e., two-thirds of more of the target area), we were able to assess the effect of visual rivalry on more realistically-sized vision multiplexing configurations (i.e., currently available smart glasses). Thus, the attenuation of binocular rivalry suppression in our results underscores the merit of using more naturalistic testing conditions in evaluating vision multiplexing HMDs.

Our results show that bilateral see-through configuration has the highest target visibility, but this design is not without behavioral costs. In Experiment 2, target visibility was significantly lower in the 3D depth condition than the 2D version, particularly for the bilateral see-through target (Fig. 5). In the natural world, double vision of background objects occurs when (1) both eyes are fixated farther or closer than these objects and (2) these objects are visible to both eyes. As the peripheral target is located closer to the subject (1 m from subject) than the fixation cross (30 m) in the 3D condition, bilateral see-through condition would produce double vision of the binocular peripheral target. These double images, also known as physiological diplopia, may capture attention and trigger a perceptual switch to be fused as a single binocular view^49^. However, because the see-through background also contains stereoscopic depth cues of farther distance, it is now unfused and may trigger a switch back towards the background. This back and forth switching effect is referred to as attentional depth switching^50–53^, and may occur repeatedly as the visual system continually registers the presence of an unfused target or background. Subjects are probably less likely to report seeing the target when their attention is captured by the unfused background, leading to the reduced target visibility in the 3D condition.

In currently available stereoscopic (bilateral) see-through HMDs such as the Hololens and 2D automotive head-up displays or HUDs (e.g., augmented reality windshield display in the Mercedes–Benz S-Class sedan and BMW i4 series), the display is viewed with both eyes and located closer than the background. As a result, the depth at which the eyes are fixated is 1 or 2 m away while the background is located at least a few meters away. Thus, the results of the bilateral see-through target in Experiment 2 imply that a similar reduction in target visibility may occur in these devices, possibly due to attentional depth switching. With depth switching, users would be required to actively ignore the double images, increasing the likelihood of fatigue and missed information. Indeed, some reported issues with bilateral see-through displays are detection errors and spatial disorientation^54^, and attentional switching costs (i.e., slower response, reduced visual search performance) in integrating information from multiple depth layers^52,53^. Unilateral displays, though less visible, do not contain stereoscopic cues of augmented information. As a result, a user reading navigational information from a unilateral see-through HMD would be less likely to experience depth switching from double vision. Moreover, the possible driver of depth switching in unilateral displays—accommodation blur—is typically suppressed by rivalry suppression^55^.

A separate but possible attentional issue in multiplexing displays is inattentional blindness. It is the apparent inability to notice significant but unexpected events in one scene when attention is allocated to another scene within the same area of the visual field^56^. Given that users of visual multiplexing devices have to switch their attentional focus between the display and background scene, inattentional blindness could have a significant effect on target detection. In fact, Apfelbaum et al.^57,58^ demonstrated that detection rates of unexpected events in monocular visual confusion (i.e., displayed superimposition of two videos on 2D screen) were reduced to half when subjects paid attention to the other scene. While our study was designed to evaluate visual rivalry in different peripheral multiplexing configurations and did not include unexpected targets, real-world uses of multiplexing displays may involve interactions between inattentional blindness and visual rivalry. For example, the binocular rivalry provoked in unilateral multiplexing configurations may further inattentional blindness, as it is much more difficult to detect an unexpected target if it is substantially suppressed from awareness. A similar situation may apply to bilateral see-through configurations, though the higher target visibility in this configuration may support the likelihood of detecting unexpected targets. Depth switching effects in this configuration, however, may interact with inattentional blindness. This is because repetitive switching between the display and background scene could pose a high amount of distraction from what the user is trying to focus at the moment (i.e., high perceptual load), which has been shown to increase inattentional blindness^59^.

These collective issues raise concerns about the practicality of peripheral multiplexing displays, but we suspect the effects may be smaller in real-world scenarios. Firstly, real-world use of multiplexing displays involves free eye movements. In Experiment 3, we found that executing eye movements (i.e., saccadic and smooth-pursuit conditions) increases target visibility for all three types of multiplexing configurations (about 20%), even when the same 3D depth condition was used. This increase may be explained by the changes in retinal images during eye movements, which interrupt the adaptation of local retinal cells^60^ and the inhibitory processes between the eyes^61^. In fact, studies have found that eye movements can induce perceptual switches favoring the suppressed image during binocular rivalry^62,63^. In other words, whenever we direct eye movements as a response to the fixation cross cue in Experiment 3, target visibility is continually supported by the interruption of rivalry suppression. A similar attenuation may apply for monocular rivalry, as it shares late visual processing mechanisms with binocular rivalry^25^. Interestingly, our results also suggest that the attenuation of rivalry suppression does not vary with the size of retinal image shifts, as target visibility was comparable between the saccadic and smooth-pursuit conditions. Future investigations could consider using a larger eye tracking region, because it is plausible that the null result may be a consequence of constraining eye movements within a small area (i.e., $5^\circ \times 5^\circ$).

Secondly, our experiments were conducted using an HMD, where there is a mismatch between accommodation and convergence, also known as the accommodation-vergence conflict^64,65^. In the real world, convergence and accommodation work together to bring objects of interest into a single, focused binocular view, whereas objects that do not correspond to the current state of accommodation and convergence will produce blurred and double images^50,51,66^. This is not the case in HMDs, where convergence cues are used to simulate different virtual distances while accommodation is fixed on optical distance about one or two meters from the eyes. As a result, the double images experienced in the HMDs are not blurred, providing a stronger cue for depth switching than would otherwise be experienced in the real world. In addition, involuntary fluctuations between the accommodational depth plane and the convergence depth plane may occur, because the state of accommodation triggers changes in convergence and vice versa^50,51,67^. These continual changes in ciliary and oculomotor muscle responses may contribute to fatigue, missed information, and further reductions in target visibility. Note, however, that the extent to which real-world conditions mitigate visual rivalry and depth switching is unclear. In the real world, the presence of accommodation blur would increase the rivalry suppression of the display when the user is looking at a distance^55^. Vestibular signals during mobility would also increase display suppression, as they increase visibility of congruent stimuli during visual rivalry^68^, which in this case would be the background motion.

What then, would be an optimal configuration for peripheral multiplexing displays? A straightforward approach would be to choose a configuration least affected by visual rivalry and depth switching. The bilateral display appears to be most resistant to visual rivalry suppression, but it remains susceptible to depth switching, albeit reduced with naturalistic viewing behavior. An alternative approach is to minimize rivalry effects in unilateral displays, and this may be achieved by manipulating the display contrast. The contrast manipulation might have to be dynamic, because further analyses of our data showed that mere increases in display contrast (i.e., unilateral opaque versus unilateral see-through) increased overall visibility but not the average duration of each period when the target was visible (Fig. S2). Some possible options are to introduce contrast transients in the display to maintain its dominance over the conflicting background area^69^, or to modulate the display contrast with the effects of vestibular signals on visual perception^68^ in mind, though the specifics of this modulation are a topic of further research.

### Supplementary Information

Supplementary Information 1.

Supplementary Information 2.

Supplementary Information 3.

Supplementary Information 4.

## Data Availability

The datasets analyzed during the current study are available at the Open Science Framework repository, 10.17605/OSF.IO/A7KFH.
