# Supplementary material for: The effect of visual rivalry in peripheral head-mounted displays on mobility
Source: Sci Rep. 2023 Nov 18;13:20199. doi: 10.1038/s41598-023-47427-8 (PMC10657352; doi:10.1038/s41598-023-47427-8)
Supplement: Supplementary file 1 — Supplementary Information 1. [file 41598_2023_47427_MOESM1_ESM.pdf]

Supplementary Information

The Effect of Visual Rivalry in Peripheral Head-mounted Displays on Mobility

Shui'er Han, Sujin Kim, and Jae-Hyun Jung

Mean visibility and suppression durations

Our results showed that target visibility varied with multiplexing configuration. To further examine the differences in target visibility, we calculated the average duration of each period when the target was visible or suppressed for each condition. In Experiment 1, we found a significant main effect of the multiplexing configuration ($F(1.23, 13.56) = 17.08$, $p = 0.001$, $\eta^2 = 0.61$; Fig S1B) on the mean visibility duration. The mean visibility duration was significantly longer for bilateral see-through configuration, compared to both unilateral opaque configuration ($t(11) = 4.06$, $p = 0.004$, $d = 1.2$) and unilateral see-through configuration ($t(11) = 4.55$, $p = 0.002$, $d = 1.3$). However, there was no noticeable difference in the mean durations between unilateral opaque and unilateral see-through configurations ($t(11) = 0.44$, $p = 0.666$, $d = 0.13$). The target was not visible for a slightly longer period (i.e., suppression duration) for the unilateral see-through configuration than the unilateral opaque configuration (i.e., averages of 3.27 and 2.59 s respectively), but the difference did not reach statistical significance ($t(11) = 1.5$, $p = 0.16$, $d = 0.4$). Thus, compared to display contrast, the choice of using a bilateral or unilateral display had a larger effect on target visibility in Experiment 1, which can be observed at the level of mean visibility and suppression durations. Lastly, the mean visibility durations did not differ across varying eccentricities ($F(2,22) = 0.58$, $p = 0.568$, $\eta^2 = 0.05$; Fig S1C). Instead, the suppression durations were increased with increasing eccentricity ($F(1.14,12.45) = 4.59$, $p = 0.049$, $\eta^2 = 0.294$; Fig S1C).

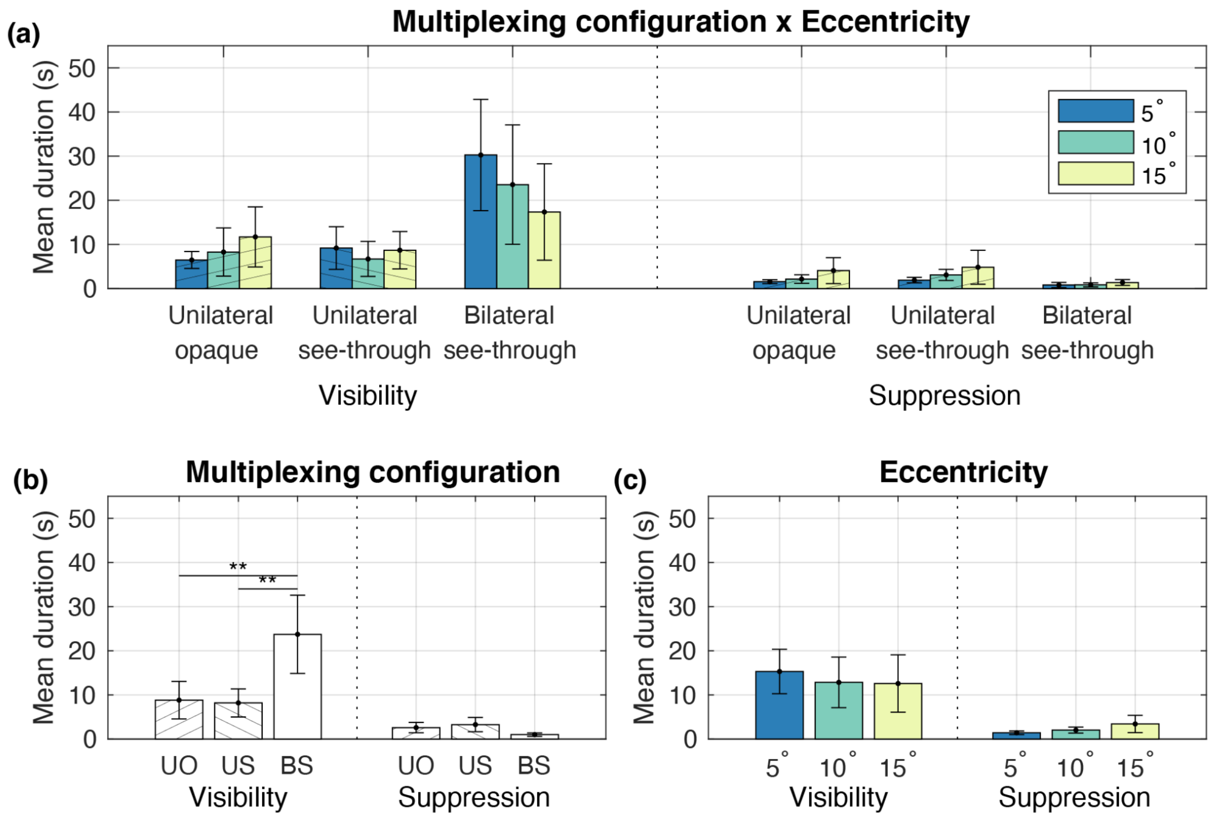

Figure S1. Mean visibility and suppression durations in Experiment 1. (a) Interaction between the multiplexing configuration and eccentricity on mean visibility and suppression durations. (b) Main effect of the multiplexing configuration. Mean visibility durations were significantly longer in bilateral see-through configuration than in unilateral opaque and unilateral see-through configurations. (c) Main effect of the eccentricity. Mean suppression durations became longer with increasing eccentricities. * UO = Unilateral opaque, US = Unilateral see-through, BS = Bilateral see-through.

In Experiment 2, mean visibility durations showed significant modulation by the multiplexing configuration ($F(1.27, 15.22) = 13.69$, $p = 0.001$, $\eta^2 = 0.53$; Fig S2B), but not by the background depth ($F(1,12) = 0.15$, $p = 0.704$, $\eta^2 = 0.01$; Fig S2C). Similar to the pattern observed in target visibility, pairwise comparisons showed that the mean visibility duration was significantly longer for bilateral see-through configuration compared to that of unilateral opaque configuration ($t(12) = 3.67$, $p = 0.006$, $d = 1.02$) and unilateral see-through configuration ($t(12) =$

3.95, p = 0.006, d = 1.1). While the mean visibility duration was 12.76 s for unilateral opaque and 8.66 s for unilateral see-through configurations, the difference between them did not reach statistical significance (t(12) = 1.95, p = 0.075, d = 0.54). Again, we observed a trend for longer suppression duration on average for unilateral see-through configuration (5.74 s) than the other multiplexing configurations (2.84 s for unilateral opaque and 3.25 s for bilateral see-through), but the trend was not statistically significant (F(1.23,14.8) = 2.9, p = 0.104, η^2 = 0.194). Mean suppression durations were also not significantly modulated by the type of depth cue (F(1,12) = 0.071, p = 0.795, η^2= 0.006).

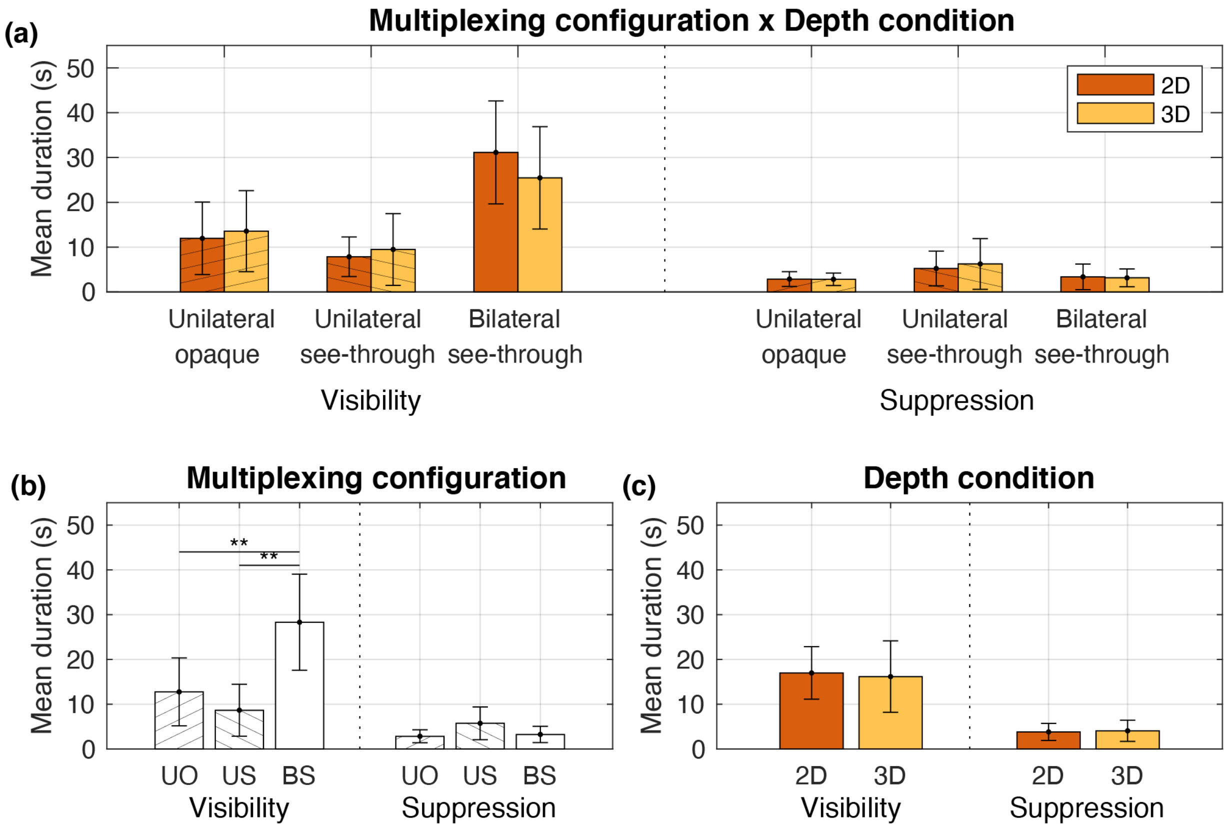

Figure S2. Mean visibility and suppression durations in Experiment 2. (a) Interaction between the multiplexing configuration and depth cue on mean visibility and suppression durations. (b) Main effect of multiplexing configuration. Bilateral see-through configuration was dominantly viewed longer on average

compared to both unilateral configurations. (c) Main effect of background depth. Both mean visibility and suppression durations were not different across background depth conditions.

In Experiment 3, the modulation in target visibility was accompanied by changes in both mean visibility and mean suppression durations. First, the multiplexing configuration yielded a significant main effect for the mean visibility duration ($F(1.4,16.74) = 15.89$, $p < 0.001$, $\eta^2 = 0.57$; Fig S3B. Bilateral target was seen for the longest duration each time it was dominant (bilateral see-through vs. unilateral opaque: $t(12) = 32.11$, $p = 0.056$, $d = 0.59$; bilateral see-through vs. unilateral see-through: $t(12) = 7.4$, $p < 0.001$, $d = 2.05$), and unilateral see-through configuration yielded shortest visibility duration on average (unilateral opaque vs. unilateral see-through: $t(12) = 3.33$, $p = 0.012$, $d = 0.92$). The mean suppression duration was also significantly different across multiplexing configurations ($F(2,24) = 12.63$, $p < 0.001$, $\eta^2 = 0.513$), in a way that the target was suppressed longer with unilateral see-through configuration than both unilateral opaque ($t(12) = 4.12$, $p = 0.004$, $d = 1.14$) and bilateral see-through ($t(12) = 3.92$, $p = 0.004$, $d = 1.09$) configurations.

Execution of eye movement significantly altered both the mean visibility ($F(2,24) = 19.41$, $p < 0.001$, $\eta^2 = 0.62$) and suppression ($F(1.12,13.48) = 8.63$, $p = 0.01$, $\eta^2 = 0.42$) durations. The target remained visible for a longer duration each time it was dominant when accompanied by the eye movements (saccade vs. fixation: $t(12) = 4.32$, $p = 0.002$, $d = 1.2$; pursuit vs. fixation: $t(12) = 5.43$, $p < 0.001$, $d = 1.51$). Furthermore, the mean visibility duration was longer when the fixation point moved continuously than moving intermittently (pursuit vs. saccade: $t(12) = 3$, $p = 0.011$, $d = 0.83$). On the other hand, the target was suppressed for the longest time when the eyes were fixated (fixation vs. saccade: $t(12) = 3.2$, $p = 0.023$, $d = 0.89$; fixation vs. pursuit: $t(12) = 2.89$, $p = 0.027$, $d = 0.8$).

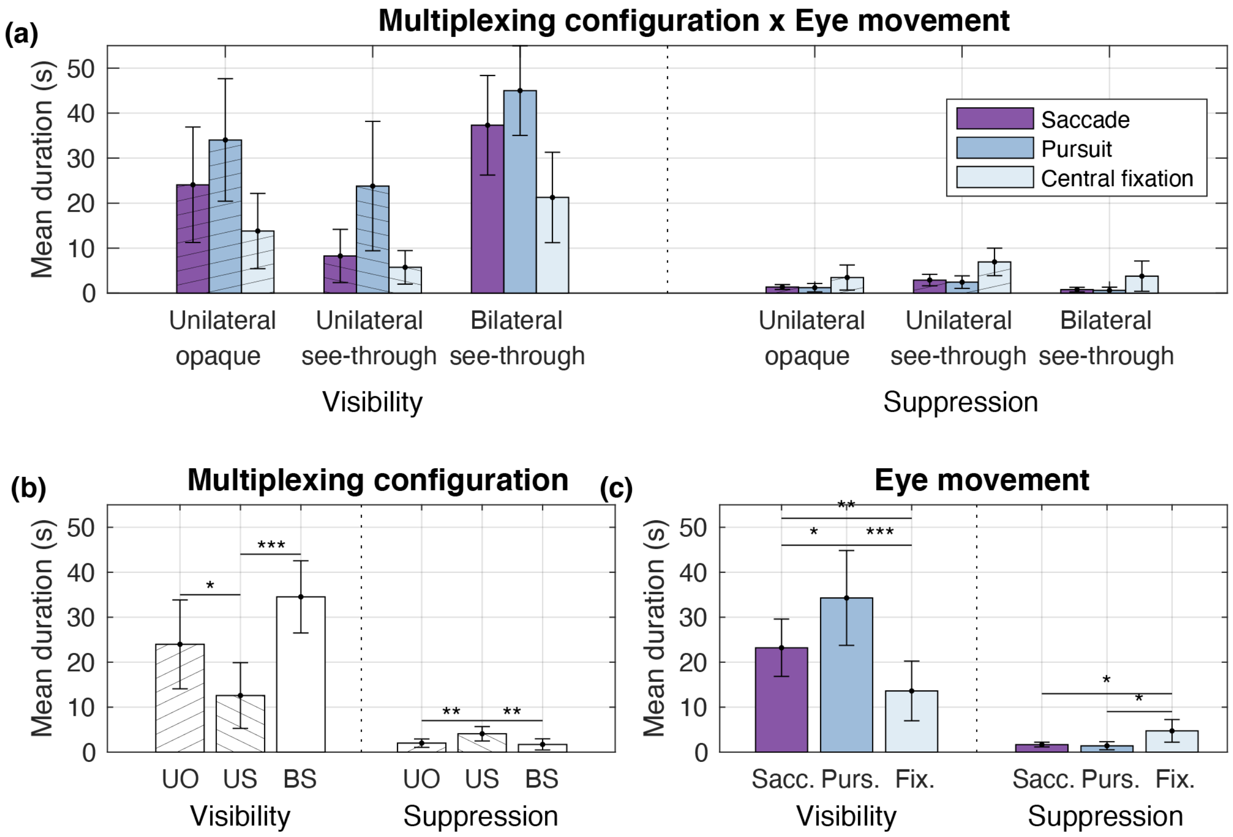

Figure S3. Mean visibility and suppression durations in Experiment 3. (a) Interaction between the multiplexing configuration and eye movement on mean visibility and suppression durations. Sacc. = Saccade, Purs. = Pursuit, Fix. = Central fixation. (b) Main effect of the multiplexing configuration. (c) Main effect of the eye movement.

Mean gaze error of all subjects

The effects of multiplexing configuration and eye movement on the gaze errors were tested with a repeated-measures ANOVA. Results showed that the main effect of eye movement was significant ($F(2,20) = 3.71$, $p = 0.043$), driven by the larger amount of gaze errors when eye movements were prompted. The main effect of the multiplexing configuration ($F(2,20) = 0.04$, $p = 0.959$) and the interaction between the two factors ($F(4,40) = 0.72$, $p = 0.584$) were not significant.

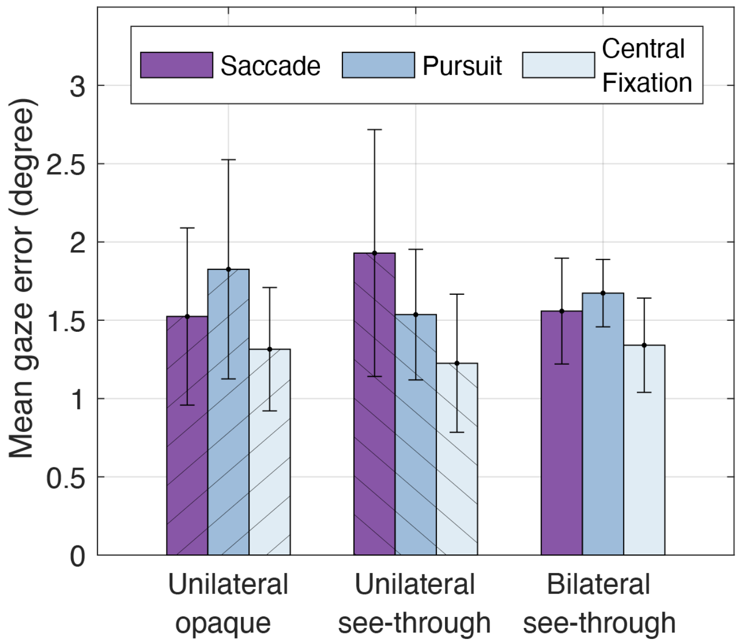

Figure S4. Group mean of gaze error. Error bars represent 95% confidence intervals.

Representative example of gaze behavior for each subject

The best gaze performances of each subject per eye movement condition is plotted below in Figure S5. As before, the positions of the fixation cross positions are represented by the solid lines and the subject's gaze positions are denoted by the dotted line. Discontinuation in the lines is are a result of eliminating invalid data such as eye blinks. As described, all subjects performed the required eye movements during the saccade, smooth-pursuit and central fixation conditions. For example, although there was some fluctuation in vertical and horizontal position for the central fixation condition (presumably due to the accommodative-vergence feedback loop), eye position mainly revolved around the 0° horizontal and vertical position. Eye movement data from subject 7 and 13 were excluded due to the technical issues during data acquisition.

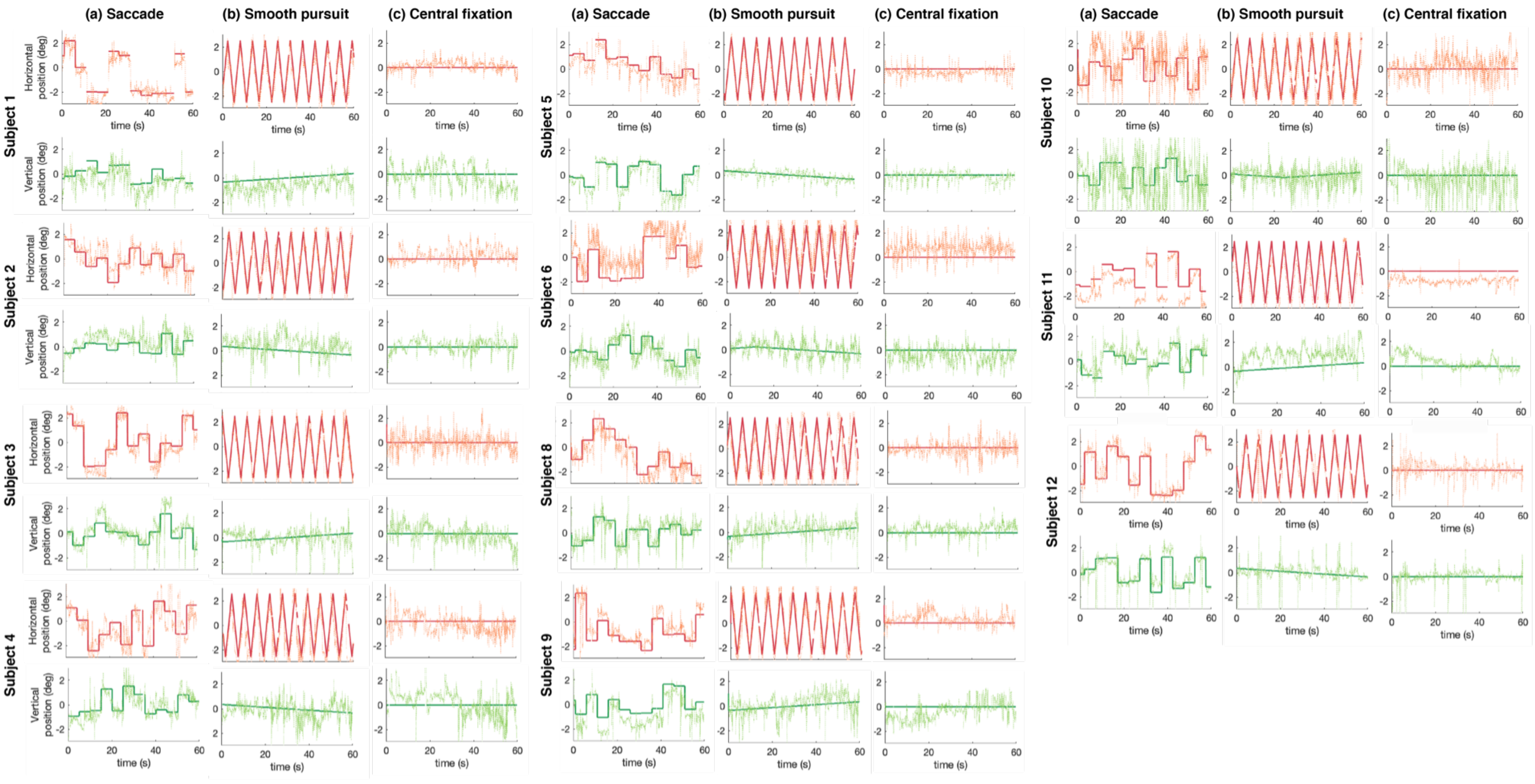

Figure S5. Representative eye movement data for each subject in Experiment 3.
